# Effect of Low-Dose (Single-Dose) Magnesium Sulfate on Postoperative Analgesia in Hysterectomy Patients Receiving Balanced General Anesthesia

**DOI:** 10.1155/2015/306145

**Published:** 2015-02-01

**Authors:** Arman Taheri, Katayoun Haryalchi, Mandana Mansour Ghanaie, Neda Habibi Arejan

**Affiliations:** ^1^Department of Anesthesiology and Pain Medicine, Tehran University of Medical Science, Tehran 19986 35696, Iran; ^2^Department of Anesthesiology, Reproductive Health Research Center, Guilan University of Medical Sciences, Guilan 14455 41656, Iran; ^3^Department of Gynecology, Reproductive Health Research Center, Guilan University of Medical Sciences, Guilan 14455 41656, Iran; ^4^Department of Internal Medicine, Metabolic Disease Research Center, Qazvin University of Medical Science, Qazvin 14816 46341, Iran

## Abstract

*Background and Aim*. Aparallel, randomized, double blinded, placebo-controlled trial study was designed to assess the efficacy of single low dose of intravenous magnesium sulfate on post-total abdominal hysterectomy (TAH) pain relief under balanced general anesthesia. *Subject and Methods*. Forty women undergoing TAH surgery were assigned to two magnesium sulfate (*N* = 20) and normal saline (*N* = 20) groups randomly. The magnesium group received magnesium sulfate 50 mg·kg^−1^ in 100 mL of normal saline solution i.v as single-dose, just 15 minutes before induction of anesthesia whereas patients in control group received 100 mL of 0.9% sodium chloride solution at the same time. The same balanced general anesthesia was induced for two groups. Pethidine consumption was recorded over 24 hours precisely as postoperative analgesic. Pain score was evaluated with Numeric Rating Scale (NRS) at 0, 6, 12, and 24 hours after the surgeries. *Results*. Postoperative pain score was lower in magnesium group at 6, 12, and 24 hours after the operations significantly (*P* < 0.05). *Pethidine* requirement was significantly lower in magnesium group throughout 24 hours after the surgeries (*P* = 0.0001). *Conclusion*. Single dose of magnesium sulfate during balanced general anesthesia could be considered as effective and safe method to reduce postoperative pain and opioid consumption after TAH.

## 1. Introduction

Postsurgical pain is one of the most important issues that could impress on postoperative peace and comfort. Abdominal hysterectomy associated with intense inflammatory response, resulting in moderate to severe postoperative pain perception [1–4].

The major goal in postoperative pain management is to minimize the dose of medications and lessen side effects, while still providing adequate analgesia [5]. Postoperative pain relief leads to earlier mobilization, shortened hospital staying, reduced hospital costs, and increased patient satisfaction [6–8].

Narcotics are the most common analgesics which are used after the surgeries. But anesthetists are always looking for replaceable methods with fewer side effects and cost [1, 2, 9, 10]. It seems adjuvant analgesics are those desirable replacements. Nowadays there have been many debates on the role of adjuvant analgesics on postoperative pain relief. One way to use adjuvant analgesics is preventive method [11–19].

Preventive analgesia is a method initiated before anesthetic procedure in order to reduce the physiological consequences of nociceptive stimulation and medical adverse effects and has been defined as an antinociceptive treatment that prevents establishment of altered central processing of afferent input from injuries. One of the intravenous adjuvant that has been shown potential in preventive analgesia is magnesium sulfate that could be administered with multiple routes or methods and one of those is preventive single low dose [1, 11–23].

Mg has been used for many years in anesthesia and cardiology as an anticonvulsant or antiarrhythmic drug. The mechanism of the analgesic effect of Mg is not clear but interference with calcium channels and N-methyl-D-aspartate (NMDA) receptor seem to play an important role. It seems that analgesic mechanism of NMDA-antagonists is made by preventing nociceptive central sensitization. Another suggesting mechanism is the role of it on reduction of catecholamine release with sympathetic stimulation, thereby decreasing peripheral nociception or the stress response to the surgery. Data illustrate that the NMDA receptor antagonists “like Mg sulfate” have an effect on pain threshold and could prevent pain perception even with low doses (preventive doses) [1, 9, 15, 18, 20, 21, 24–27].

There are few studies and some contradictions on the effect of intravenous magnesium sulfate on postoperative pain control in “total abdominal hysterectomy,” but they have been performed under different anesthetic methods. In 2008, Ryu et al. have demonstrated that coincide use of bolus and continuous infusion of intravenous magnesium sulfate during hysterectomy can reduce postoperative pain and opioid consumption under TIVA (total intravenous anesthesia) [11].

As it is confirmed, there have been some important differences between TIVA and balanced general anesthesia. In TIVA method, only intravenous anesthetic agents (hypnotics and narcotics) were used throughout the maintenance of anesthesia instead of IV anesthetics and volatile anesthetics. Whereas, balanced general anesthesia is induced with a hypnotic and Muscle relaxant agents and for the maintenance of anesthesia, volatile anesthetic, incremental doses of narcotics and muscle relaxants are used [1]. As it is obvious, opioid infusion could influence on postoperative pain score and final result.

It is necessary to mention that there are some important subjects related to the study performed by Ryu et al.: (a) different techniques of anesthesia (balanced general anesthesia versus TIVA), (b) method of “single dosage” versus “bolus + infusion” dosage, and (c) total dosage of intravenous magnesium sulfate (MgSO_4_). Moreover, Pain perception is multifactorial and depends on culture, gender, race, socioeconomic state, cognition, and previous pain memory.

Therefore, due to attention to the few studies on the effect of preventive intravenous magnesium sulfate on post-TAH pain control and other debates or differences in the present study has been conducted to determine efficiency and safety of preventive “single-low-dose” intravenous magnesium sulfate to postoperative pain relief and analgesic requirement under balanced general anesthesia in TAH.

## 2. Subjects and Method

### 2.1. Trial Design and Setting

The randomized (computerized), double-blind, placebo-controlled, clinical trial study with IRCT number of 2014012316325N1 was carried out in Obstetrics and Gynecology ward at Al-Zahra Maternity Hospital in Rasht from May 2013 to May 2014. After hospital and university ethics committee approval, the written informed consents were obtained from all patients who contributed in this study. Forty women underwent TAH operation who were in ASA (American Society of Anesthesiologists) classes I and II.

Patients who had prior abdominal surgeries, major organ system dysfunction such as kidney dysfunction, neuropathy, or myopathy, hypersensitivity or allergy to Magnesium or other products, opioid addiction, and calcium channel blockers consumption were excluded.

All cases who scheduled for elective TAH were included into the study, after approval of Hospital Ethical Committee. The trial has been fulfilled during 1 year (May 2013 and May 2014) at our maternity hospital.

### 2.2. Randomization and Interventions

Patients were randomly enrolled to one of the two groups (magnesium sulfate group versus normal saline one). Block randomization method was used to generate a random list. The magnesium group (*n* = 20) received 50 mg/kg of magnesium sulfate in 100 mL of normal saline solution “isotonic saline” as preventive dose (single low-dose), just 15 minutes before the induction of balanced general anesthesia, whereas patients in control group (*n* = 20) received 100 mL of 0.9% sodium chloride solution at the same times.

All patients received a balanced general anesthesia without premedication. The balanced general anesthesia was induced with intravenous injection of sodium thiopental 5 mg/kg, fentanyl 1 *μ*g/kg, and succinylcholine 1 mg/kg. After intubation, maintenance of anesthesia performed with balanced N_2_O/O_2_ 50%/50%, 0.5% isoflurane, atracurium 0.5 mg/kg, and fentanyl 1 *μ*g/kg. Incremental doses of fentanyl were administered, if there were any signs of sweating, lacrimation, or 20% increase in heart rate or blood pressure. Electrocardiography, heart rate monitoring, pulse oximetry, noninvasive blood pressure, and neuromuscular junction block monitoring were performed precisely. Neuromuscular blockade was antagonized with neostigmine (0.05 mg/kg) and atropine (0.02 mg/kg) at the end of operation. Heart rate and noninvasive blood pressure were recorded over 24 hours after the surgeries. In this clinical trial, patients, surgeon, outcome assessor, and ward nursing staff were blind to the group allocation. Duration of surgeries were not more than 2 hours, so there was no more fluid loss, wasting time, or abdominal stimulation. All surgeries were performed by the same gynecologist surgeon and the same anesthetist. Balanced general anesthesia was induced with the same method and minimal influence on HR and MAP (mean arterial pressure) for two groups. During intraoperative period any changes in HR (heart rate) and blood pressure (BP) were noted precisely. After the operation, patients were transferred to the recovery room and the consciousness and vital signs were evaluated until they are ready to discharge from there. In case of NRS > 4, Pethidine with dosage of 20 mg, was administered as opioid analgesia after the surgeries.

### 2.3. Data Collection and Outcomes

**Figure 1:**
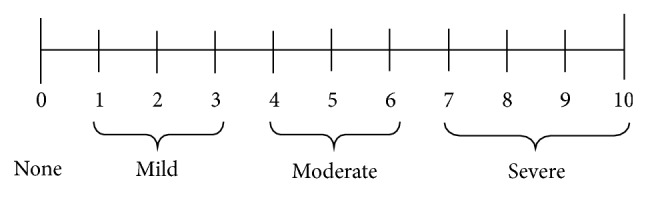
Numeric pain rating scale instructions [28].

Primary outcomes of this trial were related to pain score monitoring at 0, 6, 12, and 24 hours after the surgeries. Pain scores were evaluated by NRS (Numeric Rating Scale) with 10 cm length (starting from 0, no pain, to 10, worst pain) [28] (Figure 1).

The NRS score was recorded at emergence of anesthesia and at 6, 12, and 24 h after the surgery. The dosage and timing of analgesia were recorded at 0, 6, 12, and 24 after operation for two groups accurately. Demographic data (age, weight), duration of the surgery, and pethidine consumption were recorded over 24 hours after the surgeries exactly. According to the decision of Ethical Committee, pethidine should be administered by anesthetist, in case of NRS > 4. As well as, post-operative nausea and vomiting (PONV), hypotension and symptoms of hypermagnesemia were monitored and noted precisely at the emergence time and first day after the surgeries. Serum level of magnesium was assessed just before the bolus dose and 10 minutes after that. Hypermagnesemia was defined as serum level of magnesium more than 2.5 mEq/L. But in this study, hypermagnesemia was considered if serum magnesium level was more than 7 mEq/L (more than therapeutic dose). Any changes in heart rate (HR) and blood pressure (BP) were noted and monitored precisely during preoperative, intraoperative, and postoperative period.

### 2.4. Statistical Analysis

At least 20 patients per group were necessary, to detect a difference in 0.9 cm in pain score between two groups with an error probability of 5% and a power of 80%, assuming a standard deviation (SD) of 1 score. Data were analyzed using IBM SPSS Software Version 21. Data were shown as mean ± SD pain scores in different postoperative times between two groups which were compared using Mann-Whitney  *U*  test. Two-tiled independent *t*-test was used to compare mean age, weight, duration of surgery, and dose of pethidine consumption between two groups. A *P* value less than 0.05 has been considered as significant different.

## 3. Results

**Figure 2:**
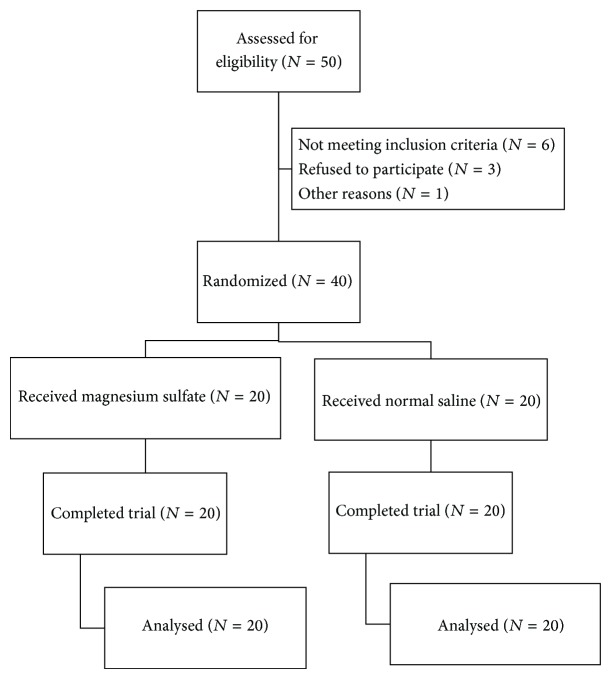
Flow of participants.

All Forty patients were divided into two groups, magnesium sulfate group (*N* = 20) and normal saline group (*N* = 20). All patients completed the trial (Figure 2). The demographic profile and duration of surgery in two groups were compared and data are depicted in Table 1. NRS (Numeric Rating Scale) with 10 cm length (starting from 0, no pain, to 10, worst pain) was evaluated at 6, 12, and 24 hours after the surgeries and compared in both groups. Pain scores decreased in magnesium sulfate group, compared with normal saline group at 6, 12, and 24 hours after the surgeries significantly (*P* < 0.05). But no significant differences were seen at the emergence time. Also, pethidine consumption in magnesium group was lower than saline group, over 24 hours after the surgeries significantly (*P* = 0.0001) (Table 2).

Preoperative mean systolic blood pressure in magnesium group was 128.8 ± 5.29 mmHg and 121.25 ± 11.10 mmHg in preoperative and postoperative period, respectively. Also, mean diastolic blood pressure was 76.0 ± 6.41 mmHg in preoperative period and 74.5 ± 5.60 mmHg in postoperative period.

In the saline group, mean systolic blood pressure was 119.25 ± 7.83 mmHg and 129.0 ± 10.71 in preoperative and postoperative times, respectively. Also, mean diastolic blood pressure was 75.25 ± 6.17 mmHg in preoperative period and 74.00 ± 4.17 mmHg in postoperative period. There was no experience of hypotension after the surgeries. None of patients experienced nausea or vomiting throughout 24 hours.

In Mg group, serum magnesium level was 2.24 ± 0.50 mEq/L before receiving magnesium and 3.43 ± 0.72 mEq/L, 10 minutes after that time. Therefore, there was not any evidence of hypermagnesemia.

## 4. Discussion

Our study has demonstrated that IV preventive dose of magnesium sulfate (50 mg kg in 100 mL of normal saline solution), just 15 minutes before the induction of balanced general anesthesia, alleviates postoperative pain throughout the first day after the abdominal hysterectomies. Furthermore, opioid (pethidine) consumption has been reduced over that time after the surgeries with no report of nausea, vomiting, hypotension, or hypermagnesemia. As intraoperative pain was measured with control of the heart rate (HR) and mean arterial pressure (MAP), any increases in those items have been considered as pain perception which needed incremental doses of opioids. There was no significant difference on NRS, just at emergence time (it means when they were transferred to the recovery room).

Pain after abdominal hysterectomy can be multifactorial. Incision pain, pain from deeper (visceral) structures, and, particularly, dynamic pain, such as during straining, coughing, or mobilizing, can be quite severe. In one study, the authors found that visceral pain dominated during the first 48 hours after hysterectomy [29].

Opioids remain the common analgesic drugs after abdominal surgeries but their adverse effects such as respiratory depression, nausea, and vomiting or hypotension make this category of drugs undesirable [1, 9].

Parenteral Mg sulfate has been used for a long time in obstetric and cardiovascular practices, but its role as an adjuvant analgesic during preoperative period specially after total abdominal hysterectomy (TAH) has been in negotiation [11, 12, 30]. As Mg sulfate is a CNS (central nervous system) depressant, sedation should be carefully monitored postoperatively. Low doses of Mg sulfate dose not interact with nondepolarizing muscle blockers (NDMB); thereby it could be its advantage. Muscle relaxant property of Mg depends on the decrease in acetylcholine release at the presynaptic level of the neuromuscular junction [1, 9, 31].

There have been numerous studies on the clinical efficacy of magnesium sulfate on postoperative pain relief that have shown conflicting results.

Wilder-Smith and colleagues used a perioperative infusion of magnesium levulinate in patients undergoing elective TAH and concluded that preoperative magnesium infusion does not improve postoperative analgesia. A small study group size and inadequate dose of magnesium might have been possible causes of this finding [10, 12, 14–16, 21, 32].

Lysakowski and colleagues in a systemic review randomized trial reached different conclusions as to whether Mg is a useful adjuvant to postoperative analgesia. Their trials do not provide convincing evidence that perioperative Mg has favorable effects on postoperative pain intensity and analgesic requirement [12]. But, in our study, results were different, and preoperative small bolus dose of Mg sulfate reduced postoperative pain scores significantly. Maybe, the source of difference was for the different method of administration.

Mavrommati and colleagues assessed the infusion of low dose Mg sulfate in hernioplasty and concluded that preventive lower bolus doses of Mg sulfate are an effective adjuvant for perioperative analgesic management [33].

Ryu and colleagues in a randomized double-blinded study assessed the effect of Mg sulfate on intraoperative anesthetic requirement and postoperative analgesia in gynecologic patients who underwent TIVA (total intravenous anesthesia) and concluded that IV Mg sulfate improves the quality of postoperative analgesia during TIVA. Results were the same of ours, but maybe the difference was for use of TIVA against balanced general anesthesia [11].

Kiran and colleagues evaluated the efficacy of single-low-dose of IV Mg sulfate for prevention of postoperative pain after inguinal surgery and concluded; it could decrease post operative pain and equivalent of rescue analgesia [34].

Hypotensive effect of Mg explained with its direct vasodilating effect through the calcium channel blockade and rarely observed with Mg up to 60 mg/kg [1, 9, 14, 26]. In the recent study, hypotensive effect of Mg sulfate has not been seen because we used even lower doses than 60 mg/kg (50 mg/kg). On the other hand, 20% increasing in MAP and HR or NRS > 4 were the indication of Pethidine administration. Throughout the first 24 hours after the surgeries, NRS was lower in Mg sulfate group compared to the control one significantly. Indeed, because for low doses of Mg, it did not intensify the action of neuromuscular depolarizing muscle blockers. Unfortunately, we did not measure ionized Mg level for some instrument restrictions but measured total serum Mg level instead. According to the results, there was not any evidence of nausea and vomiting, hypotension or hypermagnesemia. So we found that preventive doses of Mg sulfate could be useful as an adjuvant drug for TAH under balanced general anesthesia.

## 5. Conclusion

We concluded that IV preventive doses (low doses) of magnesium sulfate with dosage of 50 mg·kg^−1^ in 100 mL of normal saline solution alleviate postoperative pain throughout the first day after TAH under balanced general anesthesia significantly and reduce opioid consumption as well.

## Figures and Tables

**Table 1:** Demographic characteristics of patients in two magnesium sulfate and saline groups.

Variable	Magnesium sulfate^*^	Saline^*^	*P* value
	(*N* = 20)	(*N* = 20)	
Age (yr)	50.45 ± 5.74	51.85 ± 5.39	0.431
Weight (Kg)	76.85 ± 12.21	76.05 ± 11.05	0.829
Duration of surgery (h)	1.53 ± 0.23	1.55 ± 0.20	0.718

^*^Data are mean ± SD.

**Table 2:** The comparison of NRS mean in different time between two groups and mean postoperative analgesic consumption in two groups over 24 hours.

Variables	Magnesium sulfate^*^	Normal saline^*^	Mean difference^**^	*P* value
	(*N* = 20)	(*N* = 20)	(95% CI)	
Pain severity based on (NRS)				
Emergence time	4.30 ± 2.54	3.70 ± 0.73	0.60 (−0.59, 1.80)	0.102
6 h	6.45 ± 1.05	9.80 ± 0.62	−3.35 (−3.90, −2.80)	0.0001
12 h	5.90 ± 0.79	7.80 ± 1.11	−1.90 (−2.51, −1.29)	0.0001
24 h	4.60 ± 0.94	5.90 ± 0.45	−1.30 (−1.78, −0.82)	0.0001
Pethidine consumption (mg)	16.75 ± 18.23	68.0 ± 17.42	−51.25 (−62.67, −39.83)	0.0001

^*^Data are mean ± SD.

^**^Difference between magnesium sulfate NRS mean and saline NRS mean and 95% confidence interval of calculated mean difference.

## References

[1] Miller R. D. (2015). *Miller's Anesthesia*.

[2] Soave P. M., Conti G., Costa R., Arcangeli A. (2009). Magnesium and anaesthesia. *Current Drug Targets*.

[3] Ng A., Swami A., Smith G., Davidson A. C., Emembolu J. (2002). The analgesic effects of intraperitoneal and incisional bupivacaine with epinephrine after total abdominal hysterectomy. *Anesthesia and Analgesia*.

[4] Kim T. K., Yoon J. R. (2010). Comparison of the neuroendocrine and inflammatory responses after laparoscopic and abdominal hysterectomy. *Korean Journal of Anesthesiology*.

[5] Kehlet H., Dahl J. B. (1993). The value of ‘multimodal’ or ‘balanced analgesia’ in postoperative pain treatment. *Anesthesia and Analgesia*.

[6] de Beer J. D. V., Winemaker M. J., Donnelly G. A. E. (2005). Efficacy and safety of controlled-release oxycodone and standard therapies for postoperative pain after knee or hip replacement. *Canadian Journal of Surgery*.

[7] Recart A., Duchene D., White P. F., Thomas T., Johnson D. B., Cadeddu J. A. (2005). Efficacy and safety of fast-track recovery strategy for patients undergoing laparoscopic nephrectomy. *Journal of Endourology*.

[8] Watcha M. F., Issioui T., Klein K. W., White P. F. (2003). Costs and effectiveness of rofecoxib, celecoxib, and acetaminophen for preventing pain after ambulatory otolaryngologic surgery. *Anesthesia and Analgesia*.

[9] Hines R. L., Marchall K. E. (2012). *Stoeltings Anesthesia and Co-Existing Disease*.

[10] Bhatia A., Kashyap L., Pawar D. K., Trikha A. (2004). Effect of intraoperative magnesium infusion on perioperative analgesia in open cholecystectomy. *Journal of Clinical Anesthesia*.

[11] Ryu J.-H., Kang M.-H., Park K.-S., Do S.-H. (2008). Effects of magnesium sulphate on intraoperative anaesthetic requirements and postoperative analgesia in gynaecology patients receiving total intravenous anaesthesia. *British Journal of Anaesthesia*.

[12] Lysakowski C., Dumont L., Czarnetzki C., Tramèr M. R. (2007). Magnesium as an adjuvant to postoperative analgesia: a systematic review of randomized trials. *Anesthesia and Analgesia*.

[13] Dabbagh A., Elyasi H., Razavi S. S., Fathi M., Rajaei S. (2009). Intravenous magnesium sulfate for post-operative pain in patients undergoing lower limb orthopedic surgery. *Acta Anaesthesiologica Scandinavica*.

[14] Albrecht E., Kirkham K. R., Liu S. S., Brull R. (2013). Peri-operative intravenous administration of magnesium sulphate and postoperative pain: a meta-analysis. *Anaesthesia*.

[15] Tramèr M. R., Schneider J., Marti R.-A., Rifat K. (1996). Role of magnesium sulfate in postoperative analgesia. *Anesthesiology*.

[16] Kogler J. (2009). The analgesic effect of magnesium sulfate in patients undergoing thoracotomy. *Acta Clinica Croatica*.

[17] Paech M. J., Magann E. F., Doherty D. A., Verity L. J., Newnham J. P. (2006). Does magnesium sulfate reduce the short and long term requirement for pain relief after cesarean delivery?. *The American Journal of Obstetrics and Gynecology*.

[18] Hwang J.-Y., Na H.-S., Jeon Y.-T., Ro Y.-J., Kim C.-S., Do S.-H. (2010). I.V. infusion of magnesium sulphate during spinal anaesthesia improves postoperative analgesia. *British Journal of Anaesthesia*.

[19] Ko S.-H., Lim H.-R., Kim D.-C., Han Y.-J., Choe H., Song H.-S. (2001). Magnesium sulfate does not reduce postoperative analgesic requirements. *Anesthesiology*.

[20] Mostafa Alavi S., Baharestani B., Farsad B. F. (2011). Intraoperative magnesium sulfate can reduce narcotic requirement after coronary bypass surgery. *The Iranian Journal of Cardiac Surgery*.

[21] Koinig H., Wallner T., Marhofer P., Andel H., Hörauf K., Mayer N. (1998). Magnesium sulfate reduces intra- and postoperative analgesic requirements. *Anesthesia & Analgesia*.

[22] Levaux C., Bonhomme V., Dewandre P. Y., Brichant J. F., Hans P. (2003). Effect of intra-operative magnesium sulphate on pain relief and patient comfort after major lumbar orthopaedic surgery. *Anaesthesia*.

[23] Woolf C. J., Chong M.-S. (1993). Preemptive analgesia—treating postoperative pain by preventing the establishment of central sensitization. *Anesthesia and Analgesia*.

[24] Haryalchi K., Ghanaie M. M., Yaghoubi Y., Milani F., Faraji R. (2013). An assessment of changes in Magnesium level during gynecological abdominal surgeries. *Journal of Basic and Clinical Reproductive Sciences*.

[25] The Eclampsia Trial Collaborative Group (1995). Which anticonvulsant for women with eclampsia? Evidence from the collaborative eclampsia trial. *The Lancet*.

[26] The Magpie Trial Collaborative Group (2002). Do women with pre-eclampsia, and their babies, benefit from magnesium sulphate? The Magpie Trial: a randomised placebo-controlled trial. *The Lancet*.

[27] BTS/SIGN (2004). *British Guidline on the Manegement of Asthma*.

[28] McCaffery M., Beebe A. (1989). *Pain: Clinical Manual for Nursing Practice*.

[29] Leung C. C., Chan Y. M., Ngai S. W., Ng K.-F. J., Tsui S. L. (2000). Effect of pre-incision skin infiltration on post-hysterectomy pain—a double-blind randomized controlled trial. *Anaesthesia and Intensive Care*.

[30] Lee D. H., Kwon I. C. (2009). Magnesium sulphate has beneficial effects as an adjuvant during general anaesthesia for Caesarean section. *British Journal of Anaesthesia*.

[31] Ozcan P. E., Tugrul S., Senturk N. M. (2007). Role of magnesium sulfate in postoperative pain management for patients undergoing thoracotomy. *Journal of Cardiothoracic and Vascular Anesthesia*.

[32] Wilder-Smith C. H., Knöpfli R., Wilder-Smith O. H. G. (1997). Perioperative magnesium infusion and postoperative pain. *Acta Anaesthesiologica Scandinavica*.

[33] Mavrommati P. D., Gabopoulou Z. T., Papadimos C. N. (2004). The perioperative infusion of low doses of magnesium sulfate reduces analgesic requirements in patients undergoing abdominal hernioplasty. *Acute Pain*.

[34] Kiran S., Gupta R., Verma D. (2011). Evaluation of a single-dose of intravenous magnesium sulphate for prevention of postoperative pain after inguinal surgery. *Indian Journal of Anaesthesia*.

